# Tongue Squamous Cell Carcinoma in a European Lynx (*Lynx Lynx*): Papillomavirus Infection and Histologic Analysis

**DOI:** 10.3390/vetsci5010001

**Published:** 2018-01-02

**Authors:** Gennaro Altamura, Claudia Eleni, Roberta Meoli, Giusy Cardeti, Klaus Günther Friedrich, Giuseppe Borzacchiello

**Affiliations:** 1 Department of Veterinary medicine and Animal production, University of Naples Federico II, Via Veterinaria 1, 80137 Naples, Italy; gennaro.altamura@unina.it; 2 Istituto Zooprofilattico Sperimentale del Lazio e della Toscana ‘M. Aleandri’, Via Appia Nuova 1411, 00178 Rome, Italy; claudia.eleni@izslt.it (C.E.); roberta.meoli@izslt.it (R.M.); giusy.cardeti@izslt.it (G.C.); 3 Fondazione Bioparco di Roma, Viale del Giardino Zoologico 20, 00197 Rome, Italy; klaus.friedrich@bioparco.it

**Keywords:** bobcat, lynx, papillomavirus, squamous cell carcinoma

## Abstract

Oral squamous cell carcinoma (SCC) is a common finding in domestic and wild felids. Only two cases of oral SCC have been reported in *Lynx* species (*Lynx rufus* and *Lynx canadensis*), at mandibular and gingival sites. In this study, we describe the first report of tongue SCC in a 15 years old female European lynx *(Lynx lynx*), along with viral investigations. Necropsy and histological analysis were performed and the presence of papillomavirus (PV) infection was investigated by ultrastructural and molecular methods. The lardaceous mass at tongue level was histologically diagnosed as moderately differentiated SCC. Typical microscopical features of SCC were also found in the retropharyngeal lymph node and at the pulmonary level. Neither viral DNA by PCR, nor viral particles by transmission electron microscopy were found. Despite that PV infection is associated with Felidae, this work reports the first description of tongue SCC in Lynx species, but no evidence of PV infection, suggesting that PV may not be involved in development of SCC in bobcat species.

## 1. Introduction

Squamous cell carcinoma (SCC) is an epithelial tumour arising from both mucosal and cutaneous epithelia. SCC of the oral cavity is a common finding in the domestic cat (*Felis catus*), however it has also been diagnosed in wild felids, such as *Lynx* species at both gingival and mandibular region [1,2,3]. Feline cutaneous SCCs are very frequently associated with *Felis catus* papillomavirus type-2 (FcaPV-2) infection and the co-causative role of this papillomavirus (PV) in cancer development has been recently demonstrated [4,5]. FcaPVs DNA is rarely detectable in cat oral SCC, however their tumorigenic potential in oral cavity is still unclear [4,6]. Moreover, oral benign tumours from *Lynx* have been previously associated to PV infection and a full-length *Lynx* PV genome (*Lynx rufus* PV type-1, LrPV-1) has been isolated and cloned, however the presence of PV in oral SCC in this species has never been investigated, so far [7,8].

Here, we describe the first report of tongue SCC from *Lynx*; moreover, molecular and ultrastructural analysis for detection of PV presence was performed, but the lesion was not associated with PV infection.

## 2. Materials and Methods

### 2.1. Case Information and Histologic Analysis

A 15 years old female European lynx (*Lynx lynx)*, housed at the Zoological Garden (Bioparco) of Rome—Italy, was examined under general anaesthesia after two weeks history of dysphagia, sialorrhoea, and progressive weight loss. Examination of oral cavity revealed an extensive and infiltrating mass at the base of the tongue. As the surgical removal of the mass was not possible due to its location and infiltrating appearance, on welfare grounds, the lynx was euthanized and submitted for post-mortem examination. A complete necropsy was performed and samples of tongue, right tonsil, right retropharyngeal lymph node, lungs, liver, kidneys, and brain were collected for laboratory exams.

Samples of collected organs were fixed in 10% neutral buffered formalin and then embedded in paraffin. Sections were cut at 5 μm thick and stained with Haematoxylin and Eosin (HE) for light microscopical observation.

### 2.2. Transmission Electron Microscope (TEM) Analysis

SCC samples were prepared for negative staining using 2% (*w*/*v*) phosphotungstic acid (pH 6.6). Support 400 mesh copper grids, which were covered with a carbon reinforced plastic film, were used for the analyses. Before use, each grid was subjected to Alcian Blu staining, to ensure the grids were highly hydrophilic. Each sample (approximatively 1 g) was ground in 5 mL of sterile distilled water (20% *w*/*v*), to form a suspension and clarified by centrifugations at 3.000× *g* for 30 min and 9.000× *g* for 30 min. Eighty μL of the supernatant were ultracentrifuged in Airfuge Beckman for 20 min at 21 psi (82.000× *g*) and pelleted on a formvar-coated grid. Each grid was then placed onto a drop of 2% phosphotungstic acid (pH 6.6) for 2 min to counter-stain the grid and again excess stain removed by wicking dry. Analysis of each sample was undertaken on a Phillips EM 208, TEM at ×28.000 magnification at 80 kilovolts. Analysis time was standardized as taking 20 min. viewing of the sample grid or checking 25 grid squares whichever was shortest, to observe if any virus particles were present [9].

### 2.3. Polymerase Chain Reaction (PCR) for Detection of PV DNA

Total DNA was recovered from paraffin-embedded sections of the SCC by using DNeasy Blood & Tissue Kit (Qiagen, Hilden, Germany), according to the manufacturer’s recommendations. The obtained DNA samples were quantified by a NanoVue™ Plus spectrophotometer (GE Healthcare, Milano, Italy).

To assess the presence of PVs DNA, 50 ng of purified DNAs were subjected to PCR with AmpliTaq Gold DNA Polymerase kit (Applied Biosystems, Waltham, MA, USA) by using two different sets of degenerated primers, namely FAP59/64 and MY09/11, which are able to amplify conserved regions of the L1 viral gene from a wide range of human and animal PVs [10,11]. Positive control for FAP59/64 primers was DNA from a BPV-positive cutaneous fibropapilloma [12], one sample with no DNA template was run as negative control. For MY09/11 primer set, two PV positive bovine fibropapillomas [12], and two PV positive reptile specimens [13] were run as positive control, one normal skin sample from cat, and one sample with no template were used as negative control.

Additionally, a new primer set was designed to amplify a 147 bp sequence internal to the putative FAP59/64 amplicon from LrPV-1 L1 gene: LrPV-1-Fw: 5′-TGCTGACAATGCTCCAGACC-3′; LrPV-1-Rev: 5′-CACTGCTGGCTTTCATGAGC-3′. PCR on SCC DNA and nested PCR on 1 uL of FAP59/64 PCR product were carried out with the following amplification protocol: 95 °C for 10 min, 35 cycles of amplification at 95 °C for 30 s, 60 °C for 30 s, 72 °C for 30 s, and a final elongation step of 5 min at 72 °C. One sample with no DNA template was used as negative control. The amplification products were run by 1.5 or 2% Tris-Borate-EDTA (TBE) agarose gel electrophoresis along with a 100 bp DNA ladder and visualized by ethidium bromide stain using the ChemiDoc gel scanner (Bio-Rad, Segrate (MI), Italy).

## 3. Results

### 3.1. Gross Pathological Findings

**Figure 1:**
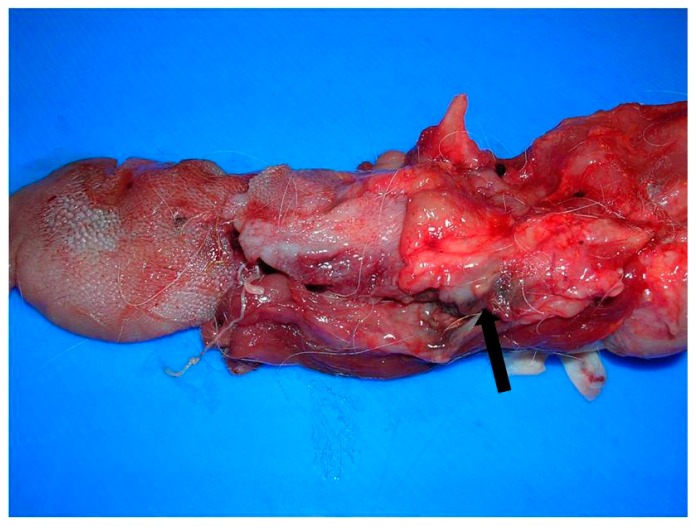
Tongue. Lardaceous mass infiltrating the base of the tongue, in particular the right side, and adjacent tissues. Necrotic-haemorrhagic area was seen (arrow).

At necropsy, a poorly demarcated 3 × 2 cm mass, infiltrated the base of the tongue, in particular the right side, involving the adjacent soft tissues, including the right retropharyngeal lymph node and the right tonsil. The mass was lardaceous in appearance with necrotic-haemorrhagic areas (Figure 1). Lungs were partially collapsed and showed numerous 2–3 mm necrotic nodules scattered in the parenchyma. Kidneys were hypotrophic, pale and with increased consistency. Mild liver degeneration, catarrhal gastroenteritis, and dilatation of the lateral cerebral ventricles were also seen.

### 3.2. Histological Findings

**Figure 2:**
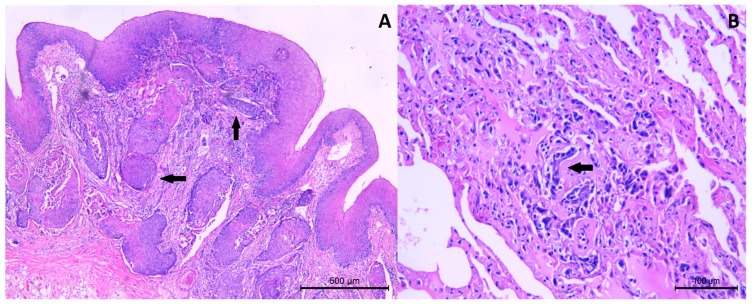
Tongue. Squamous cell carcinoma (SCC). (**A**) Neoplastic cells were arranged in cords and islands (arrows) and extended to submucosa. HE. (**B**) Lung. Metastatic SCC cells. Cords of neoplastic cells were seen within the alveolar wall (arrow). HE.

Histologically, the tongue mass was characterized by neoplastic proliferation of squamous epithelial cells originating from the mucosal epithelium. The cells were arranged in cords and nest, extended to submucosa and muscolature of the tongue, and were associated to desmoplastic response (Figure 2A). The neoplastic cells showed moderate eosinophilic cytoplasm and nuclei were characterized by high degree of pleomorfims (presence of multiple prominent nucleoli, vesicular nucleus, increased nucleus/cytoplasm ratio). Mitotic activity was moderate, ranging from 1 to 3 mitosis per high power fields (HPF), some of which were quite bizarre. Few keratin pearls were present, in particular, in the most superficial portion of the submucosa and the degree of squamous differentiation was moderate. Multifocal ulceration of mucosa and scattered lymphocytic infiltrates in the submucosa were seen. On the bases of histological features, the tumour was classified as moderately differentiated SCC, according to Broder’s grading system [14]. Massive infiltration of neoplastic cells was also detected in the right retropharyngeal lymph node and in the right tonsil, as well as in lungs (Figure 2B). In kidneys, severe membranoproliferative glomerulonephritis, multifocal lymphoplasmocytic interstitial nephritis, interstitial fibrosis, and numerous intratubular hyaline casts were observed. Diffuse centrolobular degeneration and light chronic periportal hepatitis were also seen in the liver.

### 3.3. Molecular and Ultrastructural Viral Investigations

**Figure 3:**
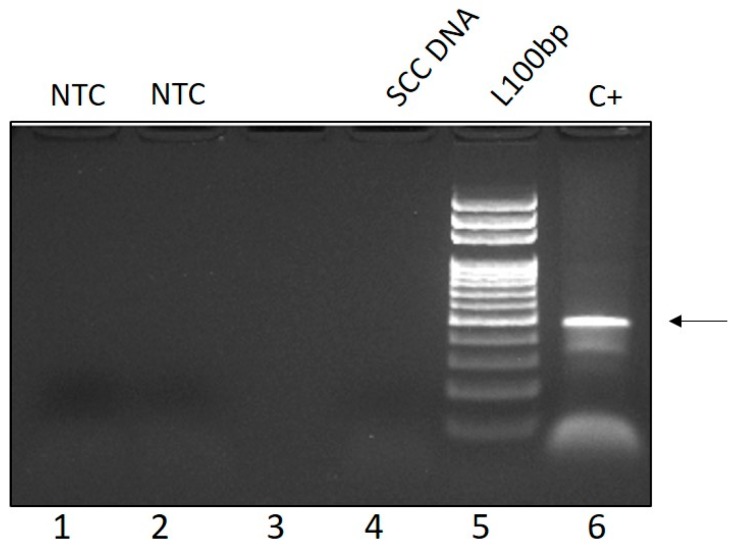
Representative PCR gel showing amplification of a 480 bp PV DNA fragment (arrow) from PV positive bovine fibropapilloma (Lane 6, C+: positive control) but not lynx tongue SCC DNA (Lane 4) with FAP59/64 primers (Lane 1, 2, NTC: no template control; Lane 3: empty well; Lane 5, L100 bp: 100 base pairs DNA ladder).

By PCR, a fragment of the expected size (480 bp) was amplified in the positive control, but not in the SCC DNA nor in the negative control by using FAP59/64 primer set (Figure 3). Similarly, with MY09/11 primers, no PV DNA was amplified in the negative controls nor in the SCC, whilst the expected band of 450 bp was detected in the positive controls. LrPV-1 specific primers did not amplify any band in the SCC sample, not even by nested PCR on FAP59/64 PCR product (data not shown). No viral particles referable to PV or any other virus were observed from the tongue samples at TEM.

## 4. Discussion

Oral SCC is among the most common tumours in the domestic feline [3]. Interestingly, it has also been reported in two different *Lynx* species, such as *Lynx rufus* and *Lynx canadensis*, at mandibular and gingival sites, respectively [1,2]. Here, we describe the first report of a tongue SCC in a felid belonging to the *Lynx* genus, namely *Lynx lynx*. Of note, the tongue is a usual location for oral SCC also in cats but less common in other animals, suggesting possible genetic and/or additional unknown factors predisposing to lingual SCC in felids with respect to other animal species [3]. A possible causative role of FcaPV-2 in the development of feline oral SCC has been recently suggested by functional studies on its E6 and E7 oncogenes and gene expression studies in vivo [4,15]. Moreover, PV infection is believed to contribute to the development of a subset of oral SCC in humans, including lingual SCC, and other animal species [16,17]. Thus, we investigated by different approaches the possible presence of PV infection in *Lynx* oral SCC. We did not detect PV’s particles by electron microscopy nor viral DNA by molecular analysis. Although this study is limited to one case, this data suggests that PV infection may not be a co-causal factor in the development of oral SCC in *Lynx lynx*. On the other hand, PV infection has been reported in oral papillomas from Florida panther (*Puma concolorcoryi*), Asian lion (*Panthera leopersica*), snow leopard (*Uncia uncia*), clouded leopard (*Neofelis nebulosa*), and bobcat (*Lynx rufus*), which may indicate that PVs could play a role in triggering oral benign lesions, but not SCC in wild felids [6,7]. Another possible hypothesis could be that PV presence is no longer detectable, since in some PV related SCC, the virus is necessary in the early stages of transformation, but then most of viral copies are lost with malignant progression [18]. Further studies are needed to clarify the possible role of PV infection in the development of oral SCC in *Lynx* species.
